# Sex Differences in Brown Adipose Tissue Function: Sex Hormones, Glucocorticoids, and Their Crosstalk

**DOI:** 10.3389/fendo.2021.652444

**Published:** 2021-04-13

**Authors:** Kasiphak Kaikaew, Aldo Grefhorst, Jenny A. Visser

**Affiliations:** ^1^ Department of Physiology, Faculty of Medicine, Chulalongkorn University, Bangkok, Thailand; ^2^ Department of Experimental Vascular Medicine, Amsterdam University Medical Centers, Location AMC, Amsterdam, Netherlands; ^3^ Department of Internal Medicine, Erasmus MC, University Medical Center Rotterdam, Rotterdam, Netherlands

**Keywords:** androgens, estrogens, glucocorticoids, progesterone, sex characteristics, sex chromosomes, steroid receptors, brown adipocytes

## Abstract

Excessive fat accumulation in the body causes overweight and obesity. To date, research has confirmed that there are two types of adipose tissue with opposing functions: lipid-storing white adipose tissue (WAT) and lipid-burning brown adipose tissue (BAT). After the rediscovery of the presence of metabolically active BAT in adults, BAT has received increasing attention especially since activation of BAT is considered a promising way to combat obesity and associated comorbidities. It has become clear that energy homeostasis differs between the sexes, which has a significant impact on the development of pathological conditions such as type 2 diabetes. Sex differences in BAT activity may contribute to this and, therefore, it is important to address the underlying mechanisms that contribute to sex differences in BAT activity. In this review, we discuss the role of sex hormones in the regulation of BAT activity under physiological and some pathological conditions. Given the increasing number of studies suggesting a crosstalk between sex hormones and the hypothalamic-pituitary-adrenal axis in metabolism, we also discuss this crosstalk in relation to sex differences in BAT activity.

## Introduction

Excessive or abnormal fat accumulation in the body causes overweight and obesity. The recent report of the World Health Organization (WHO) showed that 39.1% of adults worldwide in 2016 were overweight and 13.2% (or over 650 million) were obese, which is approximately three times as high as in 1975 (1). Obesity is a major risk for a variety of chronic diseases, including diabetes mellitus and cardiovascular diseases (CVD), mainly heart disease and stroke (1). Although the prevalence of obesity among adults is only marginally higher in women than in men (2, 3), the incidence and severity of CVD are lower in premenopausal women than in men and rise after menopause (4). Multiple determinants, such as sex hormones, sex chromosomes, physical exercise, smoking, and environmental factors, have been described to account for this sex difference in CVD and metabolic risks (5).

The major contributors to the global trend of obesity are the increased energy-rich food consumption and a physically inactive lifestyle (1), resulting in a net excess caloric intake. These excess calories are stored as triglycerides (TG) in adipose tissue which was previously considered a passive organ that stores excess energy and provides metabolic substrates for the body when needed. Nowadays, the adipose tissue is also considered an endocrine organ as it secretes various hormones called adipokines that play a role in the adaptation to different physiological and pathological conditions and that contribute to the regulation of whole-body energy homeostasis/metabolism (6).

Adipose tissue is generally categorized into two types with opposing functions: lipid-storing white adipose tissue (WAT) and lipid-burning brown adipose tissue (BAT). The latter tissue can dissipate energy (i.e., lipids) as heat instead of adenosine triphosphate (ATP), a process that is mediated by the mitochondrial uncoupling protein 1 (UCP1). Previously, it was thought that BAT was only present and active in hibernating animals, small mammals, and human infants. The traditional concept was that BAT regressed in the early years of life, leading to an absence of BAT in adults (7). Studies using positron emission tomography/computed tomography (PET/CT) in which the uptake of energy substrates indicates the metabolically active tissues, as well as UCP1 immunohistochemistry have revealed the existence of active BAT in healthy adults since 2009 (8–11). BAT is located in the cervico-supraclavicular region (between the shoulder blades), but depots are also found in the axillary, mediastinal, paravertebral, perirenal, and peri-aortic regions (12). After the rediscovery of functioning BAT in human adults, BAT has received renewed interest since its lipid oxidizing properties are considered ideal to battle the obesity pandemic.

Multiple studies have demonstrated sex differences in BAT activity. Female rodents have higher prevalence of active BAT and greater BAT mass than males (13–15). Under normal animal housing conditions at 22°C, female rats have larger mitochondria with more cristae and a higher amount of mitochondrial proteins including UCP1 in BAT than male rats (15, 16). Female BAT also displays higher protective metabolic adaptations than male BAT under energy-excess conditions. Several studies showed that when fed a high-fat diet (HFD) or a high-fat high-sugar diet, female rats increased their energy expenditure by maintaining higher amounts of thermogenic proteins such as UCP1 and PGC1α (the transcriptional coactivator of UCP1 and mitochondrial biogenesis), as well as those involved in lipid oxidation in their BAT than male rats (15, 17). Moreover, when rats were energy-deprived (i.e., received 60% of the calories of *ad libitum* fed animals for 100 days), females had a larger decline in BAT thermogenesis than males resulting in a reduced energy expenditure and preserved vital organs (18, 19).

Studies in humans also suggest that women have a higher BAT activity than men. Metabolically active BAT was detected in nearly 6% of participants in retrospective PET/CT studies (20, 21). In these studies, sex was an independent determinant of BAT activity, with women having more often detectable BAT on a fluorodeoxyglucose-PET/CT scan (20, 21). Others, however, report conflicting results, which may be because human BAT is more dispersed than the classical BAT in rodents (22). This is clear from a recent PET/CT study in non-obese adults in which it was found that women have a lower supraclavicular BAT volume but a comparable activity as men while BAT activity in the superficial dorsocervical region was more prevalent in women (23). This finding of sex differences in the distribution of active BAT adds to the complexity to study BAT function in humans.

BAT activity changes with age. Unlike the old belief, BAT mass does not decrease but even increases with age in children, a process associated with the degree of sexual maturation (24). Baseline and cold-induced BAT activity appeared greater in prepubertal girls than in boys (25). Also in young adults, cold-induced BAT activity was higher in women than in men, although the tissue density was less (26). In contrast, BAT activity in adults declines with increasing age, reflected by lipid accumulation and a decline in UCP1 expression (21, 27, 28). Interestingly, the sex dimorphism in BAT activity disappears when women become postmenopausal. This suggests that the age-related decline in circulating levels of sex hormones may contribute to this loss of BAT activity (29–31). In addition, it has been proposed that a decrease in sex hormone levels leads to a relative increase in inhibitory actions of glucocorticoids (GC) on BAT, thereby contributing to the loss of BAT activity (32), although this intriguing concept still needs to be confirmed experimentally. Species differences in BAT characteristics are summarized in **Table 1** and sex differences in BAT activity are summarized in **Table 2**.

**Table 1:** Comparison of BAT characteristics between rodents and humans.

Characteristics	Rodents	Humans
BAT distribution	Well-defined fat pads, i.e., interscapular and dorsocervical BAT	Found dispersed in many regions of the body, e.g., supraclavicular, interscapular, paravertebral/dorsal, axillary, perirenal areas
Cellular composition of BAT	Mostly homogeneous brown adipocytes	Mixture of brown, beige, and white adipocytes
ADR subtype involving BAT thermogenesis	β_3_-ADR	Likely β_2_-ADR
Effect of aging on BAT activity	Minimal decline (BAT remains active in old rodents.)	Gradual decline with age

ADR, adrenergic receptor; BAT, brown adipose tissue.

**Table 2:** Comparison of BAT features between males and females.

BAT features	Species	Findings (Females vs Males)	References
BAT mass or BAT volume (relative to body mass)	Rodents	Females > Males	(15, 16, 33–35)
		Females < Males	(31)
		No sex difference	(36)
	Humans	Females > Males	(10, 20, 28, 37)
		No sex difference	(23, 38)
BAT activity detected by PET/CT imaging	Humans	Females > Males	(10, 20, 21, 28, 37–39)
		No sex difference	(23, 40)
UCP1 protein levels	Rodents	Females > Males	(16, 17, 19, 31, 34, 35, 41, 42)
		Trend of Females > Males	(15)
*Ucp1* mRNA expression	Rodents	Females > Males	(17, 35)
		Trend of Females > Males	(15, 36, 43)
		No sex difference	(34, 41)
BAT thermogenesis or response upon adrenergic stimulation	Rodents	Females > Males	(16, 35)
		No sex difference	(15, 19)

BAT, brown adipose tissue; PET/CT, positron emission tomography/computed tomography; UCP1, uncoupling protein 1.

This review will address the mechanisms that contribute to sex differences in the regulation of BAT activity. We will focus on the roles of sex hormones in the regulation of BAT activity, i.e. thermogenesis, under physiological and pathological conditions. In addition, we will discuss the crosstalk between sex hormones and GCs in the regulation of BAT activity. Understanding the mechanisms that underlie the sex dimorphism in BAT activity will not only improve our understanding of BAT biology but will also help to better understand sex differences in metabolic diseases.

## Adipose Tissue Characteristics and Function

### Adipose Tissue Plasticity

As mentioned above, adipose tissue is principally classified into WAT and BAT. Studies have revealed the high capacity of cellular plasticity in adipose tissue. WAT can transdifferentiate into brown-like tissue by, for instance, prolonged adrenergic stimulation and acute or sustained exposure to low-temperature conditions, which are all factors that also activate BAT thermogenesis by means of stimulated lipid oxidation (44, 45). This activation process is called ‘browning’ and the resulting adipose tissue is called brite (brown-in-white) or beige adipose tissue. Of interest, the potential of cold to induce browning of WAT declines with age in mice and humans (45). In contrast, BAT can undergo ‘whitening’ as is seen, for instance, with β-adrenergic signaling impairment, chronic inflammation, high-temperature acclimation, and aging (46).

### WAT Storage Function and Distribution

WAT is not a static tissue: white adipocytes can expand through hypertrophy (increase in cell size of existing adipocytes) and/or hyperplasia (increase in number by forming new adipocytes from preadipocytes or progenitor cells) to store excess energy. Hypertrophic expansion is associated with adverse metabolic consequences because the enlarged cells exceed a maximum limit of oxygen diffusion which might lead to hypoxia and even fibrosis and inflammation, and hence insulin resistance (47–50). In contrast, hyperplastic expansion is linked with favorable metabolic outcomes and it occurs simultaneously with angiogenesis, allowing the supply of nutrients and oxygen to growing adipocytes through the newly formed blood vessels (51, 52). Of interest, angiogenesis and adipogenesis are reciprocally regulated by vascular endothelial growth factor (VEGF) and peroxisome proliferator-activated receptor-γ (PPARγ), as VEGF inhibition and loss of PPARγ activity reduce vascular formation and preadipocyte differentiation (53).

Regarding fat distribution in the body, WAT can generally be categorized into two groups by anatomical deposition: subcutaneous depots, such as anterior (axillary) and posterior (inguinal) subcutaneous depots for rodents or abdominal subcutaneous and gluteofemoral depots for humans; and visceral depots, e.g. mesenteric, gonadal, omental, and retroperitoneal depots (54, 55). Differences in WAT expansion between anatomical depots are evident in both rodent and human studies. In general, visceral obesity is associated with increased risks of metabolic complications and, therefore, measurement of waist circumference has been suggested as a reasonable proxy and indicator for the risk to develop CVDs and metabolic diseases (56–59).

Of interest, there is sexual dimorphism in fat accumulation. At an equivalent body mass index (BMI), women generally have a higher percentage of body fat than men (60–62). Moreover, women typically accumulate body fat around hips and thighs, resembling a pear-shaped body, whereas men accumulate fat around the abdomen, resembling an apple-shaped body (62–64). In other words, women, as well as female rodents, have relatively less visceral fat and more subcutaneous fat than age-matched males (65–69). This sex-dependent fat distribution becomes apparent after puberty, implying the role of sex hormones herein (70). In line with the suspected role of sex hormones, this sex difference is diminished in postmenopausal women since they gain visceral fat and their body shape converts into the male-like fat distribution (71, 72). A discussion on the effects of sex hormones on WAT function and distribution is beyond the scope of this review and has been comprehensively reviewed elsewhere (63, 73).

### BAT Thermogenesis and Metabolic Function

Under physiological circumstances, a low ambient temperature stimulates thermoreceptors in the skin and cutaneous layer that transmit sensory inputs to the preoptic area of the hypothalamus. The hypothalamic neuronal circuit then stimulates the sympathetic neurons that activate vasoconstriction in the skin and cutaneous layer in order to reduce heat loss. If rapid heat generation is required, the sympathetic neurons also induce skeletal muscle shivering and promote non-shivering thermogenesis in BAT (74). Using fluorodeoxyglucose-PET/CT scans, it has been shown that BAT activity negatively correlates with outdoor temperatures and is more prevalent during winter than other seasons (20). In humans, chronic or repeated exposure to cold, e.g. 2 hours per day for 4 weeks, increases the volume and oxidative metabolic rate of BAT (75).

**Figure 1:**
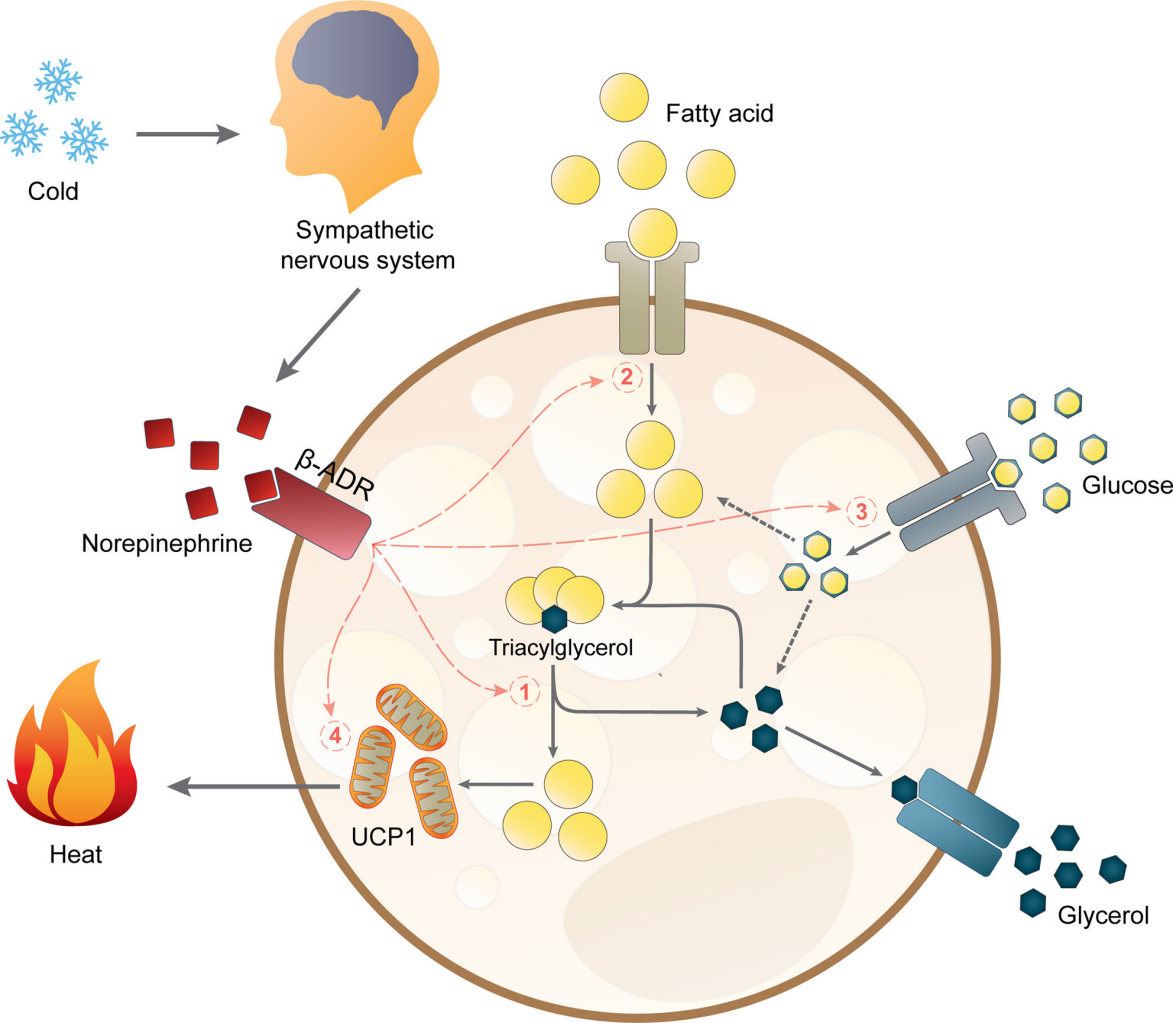
Non-shivering thermogenesis and lipid metabolism in brown adipocytes. Acute thermogenic responses by activating β-adrenergic receptors (β-ADR) which hence stimulate (1) intracellular lipolysis, (2) fatty acid uptake, and (3) glucose uptake. Altogether, these processes increase the availability of intracellular free fatty acids for thermogenesis by mitochondrial uncoupling protein 1 (UCP1). Prolonged cold exposure also induces adaptive thermogenesis by (4) upregulating *UCP1* mRNA expression.

Upon cold exposure, sympathetic nerves in BAT secrete norepinephrine to induce BAT thermogenesis *via* β-adrenergic receptors (β-ADR). In mice, β_3_-ADR has been shown to be the major β-ADR controlling BAT thermogenesis (76). However, it is still under debate if the β-ADR subtype involved in BAT activation is the same in mice and humans. A recent study suggested that β_2_-ADR is likely responsible for BAT activity in humans (77), but some studies have also shown that a β_3_-ADR agonist can increase BAT activity (78, 79). The current hypothesis on how lipid metabolism links to thermogenesis in brown adipocytes is depicted in **Figure 1**. Sympathetic activation of BAT results in intracellular lipolysis of TGs stored in lipid droplets catalyzed by adipose triglyceride lipase (ATGL), hormone-sensitive lipase (HSL), and monoacylglycerol lipase (MGL), resulting in the release of fatty acids which are the main substrate for mitochondrial respiration (80). In brown adipocytes, this respiration does not generate ATP molecules but instead will generate heat *via* the actions of UCP1 (80). Additionally, cold exposure also stimulates the uptake of fatty acids and glucose from the circulation into BAT, leading to a decline in plasma levels of free fatty acids, TG, and glucose (81). Once the fatty acids enter brown adipocytes, they will be esterified into TG and integrated into lipid droplets before they are hydrolyzed to yield the substrates for uncoupling thermogenesis (81). The importance of intracellular TG was confirmed by the impaired BAT thermogenesis in male and female ATGL-deficient mice during acute cold exposure (82). Moreover, cold exposure induces *UCP1* mRNA transcription and UCP1 protein abundance together with higher transcription and activity of crucial proteins in substrate turnover (81, 83–85). Chronic cold exposure also induces browning of WAT, as well as mRNA and protein expression of UCP1 in WAT of rodents (83, 86, 87) and humans (88). Of note, this browning is more pronounced in subcutaneous WAT than in visceral WAT (89).

Research has shown the significance of BAT in the regulation of body homeostasis through various mechanisms that include crosstalk with multiple organs [see for a comprehensive review (90)]. For instance, BAT contributes to controlling glucose homeostasis and energy balance since BAT transplantation improves insulin sensitivity, reduces body weight and WAT mass, and attenuates HFD-induced glucose intolerance (91, 92). The metabolic function of BAT thermogenesis is also supported by studies in the male UCP1-deficient mice, which show impaired glucose tolerance upon HFD feeding (93). Moreover, BAT is also an endocrine organ since it secretes various adipokines, such as the well-known adipokine adiponectin, recognized for its anti-diabetic and anti-inflammatory properties, and the more likely BAT-specific adipokines, e.g., bone morphogenetic protein 8B (BMP8B) and neuregulin-4 (NRG4) (94).

## Effects of Sex Hormones and Glucocorticoids on BAT

### Physiological Principles

The production of sex hormones is under tight regulation of the hypothalamic-pituitary-gonadal (HPG) axis (95). In brief, the gonadotropin-releasing hormone (GnRH) neurons in the hypothalamus secrete GnRH in a pulsatile fashion to induce the production and secretion of gonadotropins [follicle-stimulating hormone (FSH) and luteinizing hormone (LH)] from the anterior pituitary. In turn, gonadotropins stimulate the gonads to produce sex hormones.

In fertile women, the ovaries secrete estrogens during the follicular phase of the menstrual cycle, and estrogens and progesterone during the luteal phase. The main circulating estrogen is 17β-estradiol (E2). At target organs, estrogens typically bind to the nuclear estrogen receptors (ER), i.e., ERα and ERβ. Nuclear translocation of the estrogen-ER complex is followed by DNA binding to an estrogen response element or to other transcription factors ultimately leading to transcriptional activation or inhibition of target genes (65). In addition, estrogens can bind to the G protein-coupled estrogen receptor (GPER, previously known as GPR30) or other membrane-associated receptors to rapidly initiate non-genomic responses (96). In addition to the ovaries, progesterone is produced by the placenta during pregnancy and to some extent by the adrenal glands. The classical progesterone signaling pathway involves the nuclear progesterone receptor (PR). However, progesterone can also mediate its effects *via* non-classical pathways through receptors such as the PR membrane components (PGRMC) 1 and 2 and the membrane-associated progesterone receptors (mPR), which are members of the progestin and adipoQ receptor (PAQR) family (97).

In men, the principal circulating androgen is testosterone, produced by the testes but also in small amounts by the adrenal glands. The adrenal glands also synthesize androgenic precursors (so-called adrenal androgens), such as androstenedione, dehydroepiandrosterone (DHEA), and DHEA sulfate (98, 99). In various tissues, e.g. the prostate, the liver, and many brain regions, testosterone is converted to the very potent androgen dihydrotestosterone (DHT) by the enzyme 5α-reductase. The physiological actions of testosterone and DHT are mediated by the nuclear androgen receptor (AR). Furthermore, circulating testosterone can locally be aromatized to E2 by the enzyme aromatase (CYP19A1), thereby contributing to increased local estrogen levels. This holds true for various WAT depots, although sex differences in *CYP19A1* expression may exist (100–102). However, studies in male mice suggest that BAT does not express *Cyp19a1* and, in line, has undetectable E2 levels (102–104). Our RNA-sequencing data suggest that also in female BAT *Cyp19a1* expression is absent (43). Whether this is also true for human BAT remains to be determined, as human BAT is more heterogeneous, showing a mix of brown and white-like adipocytes (22, 105).

Studies have shown that BAT expresses the major sex hormone receptors (106), supporting the hypothesis that differences in sex hormone levels may directly contribute to the sexual dimorphism in BAT function. Given that sex hormones also regulate energy metabolism through central mechanisms, these hormones could also mediate indirect effects on BAT activity.

However, when analyzing sex differences in energy metabolism and the role of sex hormones therein, potential crosstalk with other pathways also has to be taken into account. An important crosstalk to highlight is the sex hormone-GC crosstalk as an increasing number of papers suggest that this bidirectional crosstalk may contribute to sex differences in metabolism, and potentially also in BAT activity (32, 107). GCs (cortisol in humans and corticosterone in rodents) are secreted by the adrenal cortex under the control of the hypothalamic-pituitary-adrenal (HPA) axis. GCs are involved in a broad range of physiological processes, including glucose and lipid metabolism. The effects of GCs are mediated by the nuclear glucocorticoid receptor (GR), expressed throughout the body. In addition, GCs can also signal through the mineralocorticoid receptor (MR) in certain cell types (108). Chronic exposure to elevated GC levels, as observed under stress or in Cushing’s syndrome, induces obesity not only by direct effects on adipose tissue but also at the neuroendocrine level (109). Of interest, GC synthesis and GC sensitivity show sex-specific differences (107, 110).

**Figure 2:**
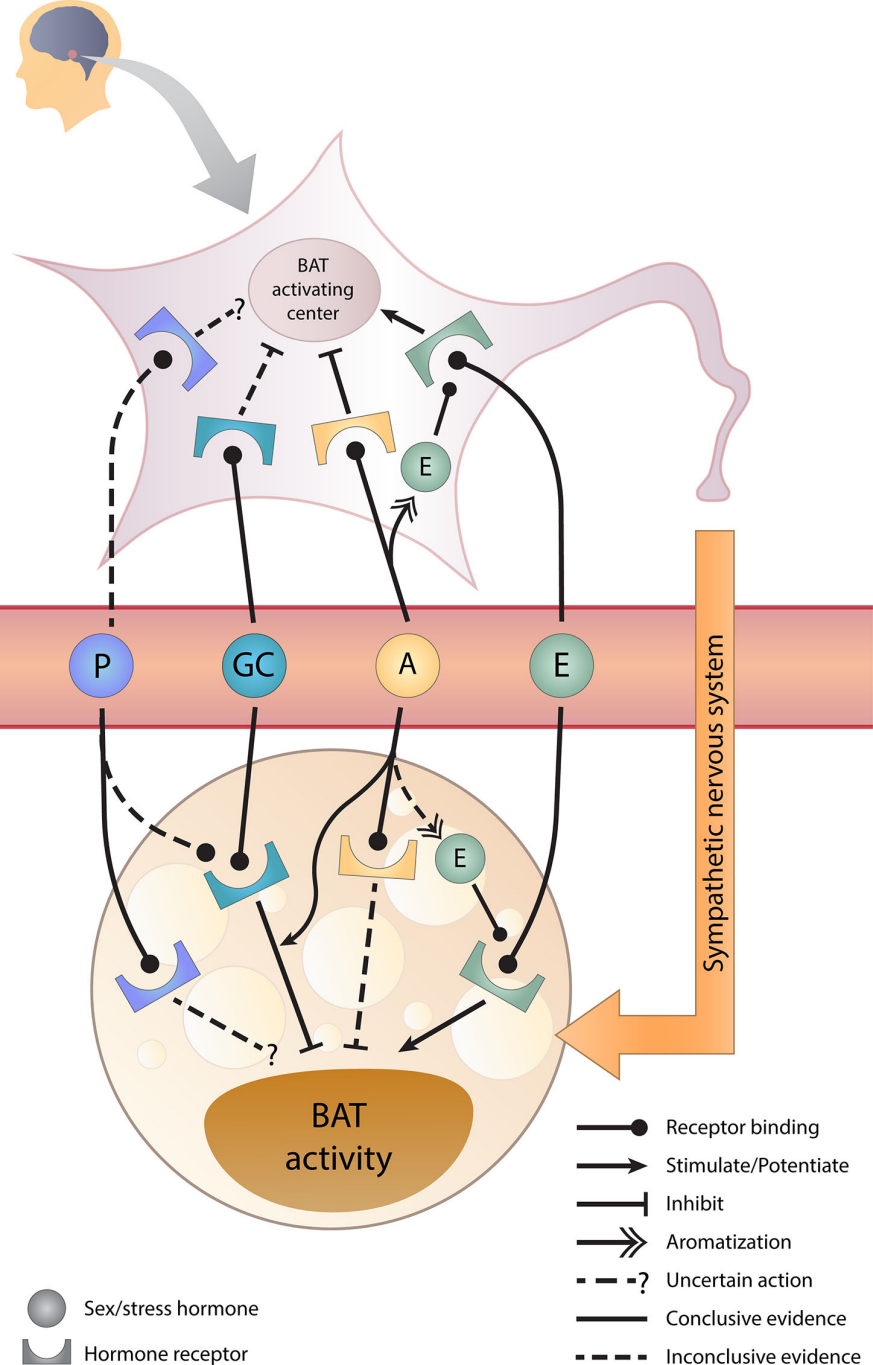
Sex hormones, glucocorticoids, and their crosstalk in BAT regulation. Estrogens stimulate whereas androgens inhibit brown adipose tissue (BAT) activity directly and indirectly *via* the brain. Glucocorticoids directly inhibit BAT activity and androgens potentiate this inhibition. However, the effect of progesterone requires further studies. A, androgens; E, estrogens; GC, glucocorticoids; P, progesterone.

The graphical overview for the effects of sex hormones, GCs, and its crosstalk on BAT activity is illustrated in **Figure 2** and the supporting evidence will be discussed in the following sections.

### Effects of Estrogens on BAT

Circulating sex hormones, particularly estrogens, are likely among the most significant regulators of BAT activity and differentiation (111). The thermogenic activity and *Ucp1* mRNA expression in BAT are reduced by ovariectomy (surgical removal of ovaries) (112, 113), while systemic administration of E2 to ovariectomized mice induces protein and mRNA expression of UCP1 in BAT (114). Moreover, estrogens, as well as cold exposure, induce whereas ovariectomy reduces transcription of BMP8B, a BAT adipokine involved in tissue remodeling for adaptive thermogenesis, in BAT of female mice (41). Using cell cultures, it has also been confirmed that E2 has a direct activating effect on brown adipocytes, for instance, by inducing the norepinephrine-induced lipolysis (a preliminary step in BAT thermogenesis) and mitochondrial biogenesis factors (115, 116). The mechanism by which estrogens promote brown adipocyte proliferation and differentiation, including *Ucp1* mRNA expression, is likely driven by ERα (117). BAT activity in whole-body ERα knockout mice has not been fully analyzed, although BAT weights were similar in both male and female ERα-knockout mice compared to wild-type mice, despite increased obesity of the knockout mice (118). Interestingly, in female mice ERα expression is two-fold higher compared to male mice and upregulated upon cold exposure (119). Furthermore, female mice with BAT-specific ERα deficiency had lower basal and cold-induced *Ucp1* expression in BAT, showed whitening of brown adipocytes, had a lower body temperature, and altered substrate metabolism in BAT during a cold challenge test compared to control mice (119). Thus, estrogens may have a direct role *via* ERα in BAT function in female mice. However, in male mice this may be less likely given that male mice have undetectable circulation estrogen levels and aromatization of testosterone in BAT of male mice is absent, as discussed above (102–104). Unfortunately, results in male BAT-specific ERα deficient mice were not reported (119). A role for ERβ in the regulation of energy metabolism is less clear. Energy metabolism seems unaltered in whole-body male ERβ-deficient mice fed a chow diet (120). However, ERβ-selective ligands appear to have anti-obesity effects. Treatment of HFD-fed mice with ERβ-selective ligands prevented the HFD-induced lipid accumulation in BAT and induced expression of mitochondrial biogenesis markers in BAT and WAT in both males and females (121, 122). Finally, whole-body GPER-knockout mice display subtle sex differences in the regulation of energy homeostasis, with more pronounced adverse effects on energy expenditure and adiposity in males. BAT of both male and female GPER-knockout mice displayed lipid accumulation and reduced mRNA expression of β_3_-ADR, while *Ucp1* gene expression was reduced in males only (123). These findings strongly indicate an important role for estrogens and their receptors in regulating thermogenic and metabolic activity of BAT. Of note, some studies demonstrated only a slight activation or no direct influence of E2 on isolated brown adipocytes (116, 124).

Apart from direct stimulating effects on brown adipocytes, estrogens also enhance BAT activity and thermogenesis *via* the brain, especially at the hypothalamic neuronal circuit, leading to an activated sympathetic nervous system (125). Intracerebroventricular administration of E2 in female rats resulted in elevated UCP1 protein content in BAT, together with a rise in the supraclavicular temperature and core body temperature (114), confirming central BAT-activating effects of estrogens. A study in ovariectomized mice illustrated the crucial potent central effects of estrogens in the regulation of BAT activity because only intracerebroventricular but not subcutaneous E2 treatment led to increased BAT *Ucp1* mRNA expression and core body temperature (126). Overall, it is evident that estrogens induce BAT activity and thermogenesis by both direct actions and indirect actions through activation of the sympathetic nervous system.

### Effects of Progesterone on BAT

The effects of progesterone on BAT have limitedly been studied and are, therefore, poorly understood. Our recent study suggests that progesterone might be involved in sex differences in BAT function (43). Analysis of the murine interscapular BAT transcriptome by RNA-sequencing identified 295 genes showing ≥2-fold sex-differential expression pattern. *In silico* analysis identified progesterone, in addition to estrogens and androgens, as one of the upstream regulators of the identified genes (43). However, *in vitro* studies of the effects of progesterone on murine brown adipocytes have provided contradictory results. While our study and others showed that progesterone reduced basal *Ucp1* mRNA expression and inhibited norepinephrine-stimulated *Ucp1* mRNA expression and lipolysis in cultured male and female brown adipocytes (43, 127), other studies showed that progesterone had a stimulatory effect on these parameters (115, 124). In part, these conflicting results can be explained by the difference in progesterone concentrations used, with high concentrations having an inhibitory effect. Additionally, it was shown that during pregnancy, when progesterone levels are high, murine BAT was less active, reflected by a reduction in mitochondrial content, thermogenic activity, and mRNA expression of *Ucp1* and other thermogenic genes (127). The reduced BAT activity during pregnancy is likely to conserve maternal energy for fetal growth (128). This inhibitory effect of progesterone on BAT thermogenic markers was confirmed in ovariectomized mice treated with progesterone (127). Information on the effects of progesterone in humans is scarce and if present indirect. Although BAT activity was not measured directly, supraclavicular temperature as a proxy of BAT thermogenesis was higher during the luteal phase compared to the follicular phase, which correlated with progesterone levels (129).

Mechanistically, it remains to be determined how the effects of progesterone on BAT are mediated. Our results suggest that when progesterone concentrations are high, part of the effects may be driven by the GR (43). Progesterone may also signal through to the MR, which was shown to be expressed in BAT (130). Effects of MR signaling are briefly discussed in the section – effects of GC on BAT. Furthermore, effects *via* progesterone membrane receptors cannot be ruled out. PGRMC1 and PGRMC2 both play a role in adipose tissue metabolism but appear to have opposing roles. Male adipose-specific PGRMC1 knockout mice are less prone to HFD-induced lipid accumulation in BAT (131), while male and female adipose-specific PGRMC2 knockout mice are defective in cold-induced thermogenesis (132). Thus, while it is clear that E2 stimulates BAT activity, the role of the ‘other’ female sex hormone progesterone in BAT physiology requires more research.

### Effects of Androgens on BAT

In contrast to estrogens, the effects of androgens on BAT activity are more difficult to unravel. *In vitro* studies suggest that androgens reduce BAT activity. In primary brown adipocytes of rodents, testosterone inhibited mitochondrial biogenesis, brown adipocyte differentiation, and norepinephrine-induced lipolysis (115, 116, 124). In immortalized mouse brown adipose cells, DHT (a nonaromatizable androgen) dose-dependently inhibited differentiation, isoproterenol-stimulated *Ucp1* mRNA expression, and mitochondrial respiration, and these findings were confirmed in explants of mouse BAT (133). However, direct *in vivo* effects of androgens appear controversial. Orchiectomy (surgical removal of testes) increased mRNA and protein expression of UCP1 in murine BAT, together with an elevated body temperature (134, 135). Yet, some studies have shown that prolonged exposure to DHT in orchiectomized male mice or in female mice did not reduce *Ucp1* mRNA expression in BAT (136–138). Furthermore, AR-knockout male mice developed late-onset obesity and displayed a reduction in the expression of thermogenic genes in BAT (139). This latter study also describes the presence of an androgen response element in the *Ucp1* promoter and showed a stimulation of an *Ucp1* promoter construct by DHT (139). Clinical studies suggest that changes in androgen levels may have an opposite effect in women and men. Hyperandrogenism in women, as in the prevalent disorder polycystic ovary syndrome (PCOS), and hypogonadism in men are both associated with abdominal obesity and obesity-related disorders (140). Compared to controls, women with PCOS have been suggested to have lower BAT activity, based on a lower supraclavicular skin temperature (141). In androgen-induced mouse models of PCOS, we and others observed that androgen excess reduced the mRNA expression of *Ucp1* and other genes involved in mitochondrial function in BAT, likely contributing to a lower body temperature (41, 142, 143).

Similar to estrogens, in addition to direct effects, androgens can have indirect effects on BAT through central mechanisms. Chronic DHT exposure in female mice reduced hypothalamic leptin sensitivity, which blunted leptin-induced BAT thermogenesis through changes in the melanocortin system (137). Whole-body AR knockout male mice also develop leptin resistance (144).

Importantly, it should be noted that part of the androgen effects could be explained by locally increased estrogen levels due to intratissue aromatization of androgens, particularly in WAT and the brain (145). Thus, part of the observed effects of testosterone may in fact be ER-mediated. A recent study showed that the testosterone-induced reduction of white fat mass in obese hypogonadal male mice requires ERα expression in the brain (104). Hence, it seems likely that *in vivo* androgens affect BAT function through central mechanisms, likely through central estrogen signaling, although the latter requires more detailed studies.

### Effects of Glucocorticoids on BAT

GCs are well known to have metabolic effects, and GC excess leads to obesity in both rodents and humans (109). Several studies in rodents have shown that upon chronic GC exposure BAT thermogenesis is inhibited. Studies from our laboratory showed that corticosterone treatment resulted in lipid accumulation and a decrease in basal and norepinephrine-induced UCP1 mRNA and protein content in BAT of both male and female mice (36, 146, 147). These effects were confirmed by others in a study in which male rats were treated with corticosterone for 21 days (148). In *in vitro* differentiated brown adipocytes, GCs reduced the norepinephrine-stimulated *Ucp1* mRNA expression (147, 149). GC inhibition of BAT activity is likely mediated through the GR since a GR-specific antagonist reversed the inhibitory effects of GC on *Ucp1* mRNA expression in mice (150–152). However, it has to be kept in mind that GC can also signal through the MR, which is also expressed in BAT (130). Activation of the MR in a mouse brown adipocyte cell line stimulates the differentiation of pre-adipocytes into mature adipocytes (153), and suppresses thermogenic activity by reducing isoproterenol-stimulated *Ucp1* transcription (154). In male mice, adipose tissue-specific MR deficiency prevents lipid accumulation in BAT upon HFD treatment (155). MR antagonist treatment also resulted in browning of WAT in diet-induced obese female mice (156). In agreement, in a small study of healthy male and female volunteers, treatment with a MR antagonist for two weeks increased BAT activity and volume (157). Whether these MR effects are mediated by GC may depend on the intratissue availability of GCs, discussed below.

Some recent studies also present contradictory data on GR signaling in BAT. GC treatment only reduced the total mRNA and protein content of UCP1 in BAT when male mice were housed at thermoneutrality (30°C) while total UCP1 protein content was not affected when mice were housed under standard housing conditions (21°C), but the mice developed obesity to a similar extent at both temperatures (158). This reported difference in GC effects on UCP1 expression can in part be explained by the way UCP1 levels were expressed, i.e. per μg protein or per total depot weight. Interestingly, in UCP1-deficient mice, GC-induced obesity was not worsened compared to wild-type mice (158), suggesting UCP1-independent effects of GCs on BAT function. Furthermore, based on a study analyzing BAT-specific GR knockout (GR^BATKO^) male mice, the role of GR in BAT function is debatable, as GR^BATKO^ mice did not differ from wild-type mice in terms of BAT thermogenesis and HFD-induced metabolic consequences (159). It should be noted that in the GR^BATKO^ male mice the HPA axis was not affected and that these mice had normal corticosterone levels. Interestingly, adrenalectomy resulted in increased BAT activity in obese male mice (160). Adrenalectomy also resulted in a differential effect on substrate uptake by BAT, as adrenalectomy in male mice abolished the circadian rhythm of glucose uptake but had no effect on its rhythms in fatty acid uptake (161). Although these studies might underscore the effects of endogenous GCs on BAT, adrenalectomy has effects beyond a reduction in GC levels as it also leads to reduced catecholamines and elevated adrenocorticotropic hormone (ACTH) levels, which have been shown to induce BAT activity in male mice (147).

Although studies are limited, also in humans GCs may inhibit BAT activity. In Cushing’s syndrome, characterized by hypercortisolism due to ACTH-secreting pituitary tumors or cortisol-secreting adrenal tumors, increased lipid accumulation occurs in the supraclavicular fat depot (162). Furthermore, prolonged GC treatment resulted in significantly fewer patients (men and women) with detectable BAT compared to controls (163), and lower cold-induced BAT activity (42). However, opposite effects were observed when comparing acute and chronic GC exposure, because acute GC treatment was shown to induce BAT activity as assessed by the supraclavicular temperature in male volunteers (163, 164). Furthermore, species difference in GC regulation of BAT has been demonstrated since GC was shown to inhibit UCP1 expression in cultured brown adipocytes of male mice whereas the opposite effect was observed in cultured human brown adipocytes (163).

The effects of GCs also depend on its intratissue concentration, which is regulated by the enzymes 11β‐hydroxysteroid dehydrogenase type 1 (11β‐HSD1) and 11β‐HSD2. 11β‐HSD1 converts the inactive GC isoform, cortisone in humans or 11‐dehydrocorticosterone in rodents, into the bioactive GC isoform, cortisol in humans or corticosterone in rodents, whereas 11β‐HSD2 inactivates the intratissue GCs (165). The GC-inactivating enzyme 11β‐HSD1 is mostly expressed in the classical MR-target tissues, such as kidneys, while the GC-activating enzyme 11β‐HSD1 is highly expressed in most metabolic tissues including BAT (166, 167). Thus, 11β‐HSD1 contributes to the intratissue availability of GC and thereby is an important factor influencing GC effects on BAT activity. Lipid accumulation in BAT upon GC treatment and aging was strongly diminished in 11β‐HSD1-deficient male mice (167). In HFD-fed male mice, treatment with an 11β-HSD1 inhibitor increased *Ucp1* mRNA expression and also reduced lipid accumulation in BAT (168). Since GCs can stimulate the expression of 11β‐HSD1, thereby increasing GC exposure to impair BAT function (158), pharmacological inhibition of 11β‐HSD1 has been proposed as a target in the treatment of obesity.

Altogether, these studies suggest that the detrimental effects of GC on BAT activity become visible under conditions where GC levels are elevated, such as under chronic stress or prolonged systemic GC administration. Hypercortisolism may also affect other endocrine systems, such as the HPG axis, and thereby sex hormone levels.

### Sex Hormone-Glucocorticoid Crosstalk in BAT

It is well-accepted that the HPA axis exhibits some sexually dimorphic features. For example, many stress-related disorders, such as anxiety and depressive disorders, are more prevalent and severe in women than in men (169). Female rats also show higher basal levels and stress-induced levels of neuroendocrine responses of the HPA axis, including corticosterone and ACTH, than male rats. Interestingly, baseline and stress-induced levels of corticosterone and ACTH were indistinguishable between the sexes after gonadectomy (170, 171), implicating a role for sex hormones in the regulation of the HPA axis. A sex-dependent bidirectional crosstalk between the HPA axis and sex hormones in metabolism has been demonstrated and proposed to play a role in the developmental misprogramming of metabolism (172). Whether such a crosstalk also plays a role in the regulation of BAT activity is less clear but we recently showed that treatment of mice with corticosterone elevated the expression of GR-target genes *Fkbp5* and *Tsc22d3* more profoundly in BAT of male than of female mice while it induced BAT whitening and reduced *Ucp1* mRNA expression in BAT to a similar extent in both sexes (36). Another study, using a lower concentration of corticosterone, also reported a negative effect of GCs on BAT, which was more pronounced in male than in female mice (135). Interestingly, the corticosterone-induced lipid accumulation in BAT of mice was absent after orchidectomy, suggesting an androgen-dependent effect of GC on BAT (135). Furthermore, ovariectomy did not sensitize BAT to glucocorticoids, but DHT treatment of ovariectomized mice did (135). Also in an *in vitro* study, DHT was shown to potentiate GC-induced GR signaling in brown adipocytes, as illustrated by an upregulation in transcriptional levels of the GR-responsive genes *Fkbp5*, *Tsc22d3*, and *Mt2a* (138). Sex hormones may also contribute to GC intratissue availability. It has been shown that androgens increase whereas estrogens decrease 11β‐HSD1 expression and activity in WAT (107, 138). Interestingly, studies using human primary white adipocytes suggest that the effect of sex hormones on 11β‐HSD1 expression may be sex-specific (173). Of interest, postmenopausal women have higher 11β‐HSD1 activity in their adipose tissue (174), underscoring the physiological relevance of the role of estrogens on 11β‐HSD1. Whether sex hormones also regulate 11β‐HSD1 expression in BAT remains to be determined. These studies suggest that a crosstalk between androgens and GCs in the regulation of BAT activity may exist. It also suggests that changes in the balance of sex hormone levels and glucocorticoid levels may impact BAT activity. This could be relevant upon aging in women when sex hormone levels decline, thereby reinforcing the inhibitory effect of GC on BAT.

### Sex Hormone-Independent Factors Contributing to Sex Differences in BAT Activity

Sex differences in metabolism can be explained by differences in the levels of sex hormones between the sexes. This difference is driven to a large extent by the difference in sex chromosomes. However, this genetic difference also results in sex-hormone independent effects. For instance, random X-inactivation in female cells and genomic imprinting of autosomes have been shown to contribute to sex-specific gene expression (175). Indeed, cultured primary brown adipocytes, isolated and differentiated from BAT of male and female mice, maintained a sex-differential expression profile even though they were cultured and differentiated under the same standard culture conditions (43). In addition, human adipocytes differentiated from pre-adipocytes isolated from female perirenal fat had a higher *UCP1* mRNA expression level than those isolated from male perirenal fat despite similar culture conditions (176).

The contribution of this intrinsic genetic difference in the regulation of BAT differentiation and activity has not been studied in great detail. In humans, dissecting the effects of genetic sex and sex hormones is difficult. In disorders of sex differentiation, an altered number of X or Y chromosomes is often associated with abnormal gonadal differentiation and function (177). So far, only the use of the four core genotypes (FCG) mice has provided a tool to study effects of genetic sex and gonadal function separately. The FCG is a mouse model in which the Y-chromosomal *Sry* gene that functions as a testis-determining factor is relocated to an autosome, allowing the generation of four types of mice: XX mice with ovaries, XX mice with testes, XY mice with testes, and XY mice with ovaries (175). In young gonadectomized FCG mice, BAT *Ucp1* expression tended to be suppressed in the presence of the Y chromosome. However, the existing gonads (testes or ovaries) were more influential than the sex chromosomes, as the orchiectomized XX or XY mice had a slightly but significantly higher BAT *Ucp1* mRNA expression than the ovariectomized XX or XY mice (178). Although further studies are needed, this study suggests that gonadal hormones have a more prominent role than sex chromosomes in the regulation of BAT activity.

Another factor that may contribute to sex differences in metabolism is epigenetic programming. Epigenetic programming has been shown to modulate BAT and WAT activity through several mechanisms, such as DNA methylation and histone modifications. Mouse models in which epigenetic mechanisms were inhibited or stimulated displayed changes in the transcriptional control of BAT and hence BAT thermogenesis (179). Furthermore, sex-specific epigenetic marks in adipose tissue were identified in the effects of early-life social disadvantage on adulthood BMI (180). However, a role in sex-differential control of BAT thermogenesis remains to be determined. Intriguingly, a single dose of testosterone administration in the neonatal period of female mice was sufficient to induce whitening of BAT and downregulate *Ucp1* and other BAT-specific gene transcriptional levels at adulthood (181). Whether this involves epigenetic programming remains to be determined. However, it does show that early life events can have lifelong effects on BAT activity and contribute to sex differences in BAT activity.

## Concluding Remarks

Studies in rodents show that sex hormones regulate BAT activity in a sex-specific manner through direct and indirect mechanisms. Estrogens induce a stimulatory effect on BAT activity, adding to the healthier metabolic actions of estrogens in females. Androgens appear to have an inhibitory effect, while the actions of progesterone on BAT function require further research. The crosstalk between sex hormones and GCs adds to the mechanisms that control sexually dimorphic BAT activity. This crosstalk also illustrates that tipping the balance in sex hormones and GC levels, but likely other factors as well, alters the effect of each of these hormones on BAT. In that respect, more knowledge about mechanisms that regulate intracellular availability and sensitivity of these hormones is warranted as these may contribute to the sex-specific sensitivity of BAT to sex hormones and GCs. Studies in humans suggest comparable effects but require further studies. Analysis of BAT activity under pathophysiological conditions may aid to gain a better understanding.

Given the proposed role of BAT as a target to battle obesity, changes in sex hormone levels may be one of the mechanisms contributing to sex differences in BAT physiology and thereby sex differences in the onset and development of obesity-related disorders.

## Author Contributions

KK and JV drafted the manuscript. All authors contributed to the article and approved the submitted version.

## Conflict of Interest

The authors declare that the research was conducted in the absence of any commercial or financial relationships that could be construed as a potential conflict of interest.

## References

[1] World Health Organization. Obesity and overweight - Fact sheets. World Health Organization (2020). Available at: https://www.who.int/en/news-room/fact-sheets/detail/obesity-and-overweight [Accessed November 20, 2020].

[2] Hales CM Carroll MD Fryar CD Ogden CL. Prevalence of Obesity and Severe Obesity Among Adults: United States, 2017-2018. NCHS Data Brief No. 360. (2020). Available at: https://www.cdc.gov/nchs/products/databriefs/db360.htm [Accessed November 20, 2020]. 32487284

[3] Malik VS Willet WC Hu FB. Nearly a decade on - trends, risk factors and policy implications in global obesity. Nat Rev Endocrinol (2020) 16(11):615–6. 10.1038/s41574-020-00411-y PMC7461756 32873971

[4] Virani SS Alonso A Benjamin EJ Bittencourt MS Callaway CW Carson AP. Heart Disease and Stroke Statistics-2020 Update: A Report From the American Heart Association. Circulation (2020) 141(9):e139–596. 10.1161/CIR.0000000000000757 31992061

[5] Colafella KMM Denton KM. Sex-specific differences in hypertension and associated cardiovascular disease. Nat Rev Nephrol (2018) 14(3):185–201. 10.1038/nrneph.2017.189 29380817

[6] Scheja L Heeren J. The endocrine function of adipose tissues in health and cardiometabolic disease. Nat Rev Endocrinol (2019) 15(9):507–24. 10.1038/s41574-019-0230-6 31296970

[7] Nedergaard J Bengtsson T Cannon B. Unexpected evidence for active brown adipose tissue in adult humans. Am J Physiol Endocrinol Metab (2007) 293(2):E444–52. 10.1152/ajpendo.00691.2006 17473055

[8] van Marken Lichtenbelt WD Vanhommerig JW Smulders NM Drossaerts JM Kemerink GJ Bouvy ND. Cold-activated brown adipose tissue in healthy men. N Engl J Med (2009) 360(15):1500–8. 10.1056/NEJMoa0808718 19357405

[9] Virtanen KA Lidell ME Orava J Heglind M Westergren R Niemi T. Functional brown adipose tissue in healthy adults. N Engl J Med (2009) 360(15):1518–25. 10.1056/NEJMoa0808949 19357407

[10] Cypess AM Lehman S Williams G Tal I Rodman D Goldfine AB. Identification and importance of brown adipose tissue in adult humans. N Engl J Med (2009) 360(15):1509–17. 10.1056/NEJMoa0810780 PMC2859951 19357406

[11] Zingaretti MC Crosta F Vitali A Guerrieri M Frontini A Cannon B. The presence of UCP1 demonstrates that metabolically active adipose tissue in the neck of adult humans truly represents brown adipose tissue. FASEB J (2009) 23(9):3113–20. 10.1096/fj.09-133546 19417078

[12] Rogers NH. Brown adipose tissue during puberty and with aging. Ann Med (2015) 47(2):142–9. 10.3109/07853890.2014.914807 24888388

[13] Law J Bloor I Budge H Symonds ME. The influence of sex steroids on adipose tissue growth and function. Horm Mol Biol Clin Investig (2014) 19(1):13–24. 10.1515/hmbci-2014-0015 25390013

[14] Kim SN Jung YS Kwon HJ Seong JK Granneman JG Lee YH. Sex differences in sympathetic innervation and browning of white adipose tissue of mice. Biol Sex Differ (2016) 7:67. 10.1186/s13293-016-0121-7 27990249 PMC5148917

[15] Rodriguez AM Quevedo-Coli S Roca P Palou A. Sex-dependent dietary obesity, induction of UCPs, and leptin expression in rat adipose tissues. Obes Res (2001) 9(9):579–88. 10.1038/oby.2001.75 11557839

[16] Rodriguez-Cuenca S Pujol E Justo R Frontera M Oliver J Gianotti M. Sex-dependent thermogenesis, differences in mitochondrial morphology and function, and adrenergic response in brown adipose tissue. J Biol Chem (2002) 277(45):42958–63. 10.1074/jbc.M207229200 12215449

[17] Choi DK Oh TS Choi JW Mukherjee R Wang X Liu H. Gender difference in proteome of brown adipose tissues between male and female rats exposed to a high fat diet. Cell Physiol Biochem (2011) 28(5):933–48. 10.1159/000335807 22178945

[18] Valle A Catala-Niell A Colom B Garcia-Palmer FJ Oliver J Roca P. Sex-related differences in energy balance in response to caloric restriction. Am J Physiol Endocrinol Metab (2005) 289(1):E15–22. 10.1152/ajpendo.00553.2004 15701677

[19] Valle A Garcia-Palmer FJ Oliver J Roca P. Sex differences in brown adipose tissue thermogenic features during caloric restriction. Cell Physiol Biochem (2007) 19(1-4):195–204. 10.1159/000099207 17310113

[20] Ouellet V Routhier-Labadie A Bellemare W Lakhal-Chaieb L Turcotte E Carpentier AC. Outdoor temperature, age, sex, body mass index, and diabetic status determine the prevalence, mass, and glucose-uptake activity of 18F-FDG-detected BAT in humans. J Clin Endocrinol Metab (2011) 96(1):192–9. 10.1210/jc.2010-0989 20943785

[21] Brendle C Werner MK Schmadl M la Fougere C Nikolaou K Stefan N. Correlation of Brown Adipose Tissue with Other Body Fat Compartments and Patient Characteristics: A Retrospective Analysis in a Large Patient Cohort Using PET/CT. Acad Radiol (2018) 25(1):102–10. 10.1016/j.acra.2017.09.007 29108812

[22] Leitner BP Huang S Brychta RJ Duckworth CJ Baskin AS McGehee S. Mapping of human brown adipose tissue in lean and obese young men. Proc Natl Acad Sci USA (2017) 114(32):8649–54. 10.1073/pnas.1705287114 PMC5559032 28739898

[23] Fletcher LA Kim K Leitner BP Cassimatis TM O’Mara AE Johnson JW. Sexual Dimorphisms in Adult Human Brown Adipose Tissue. Obes (Silver Spring) (2020) 28(2):241–6. 10.1002/oby.22698 PMC6986330 31970907

[24] Gilsanz V Smith ML Goodarzian F Kim M Wren TA Hu HH. Changes in brown adipose tissue in boys and girls during childhood and puberty. J Pediatr (2012) 160(4):604–9 e1. 10.1016/j.jpeds.2011.09.035 22048045 PMC3307823

[25] Malpique R Gallego-Escuredo JM Sebastiani G Villarroya J Lopez-Bermejo A de Zegher F. Brown adipose tissue in prepubertal children: associations with sex, birthweight, and metabolic profile. Int J Obes (Lond) (2019) 43(2):384–91. 10.1038/s41366-018-0198-7 30185921

[26] Martinez-Tellez B Sanchez-Delgado G Boon MR Rensen PCN Llamas-Elvira JM Ruiz JR. Distribution of Brown Adipose Tissue Radiodensity in Young Adults: Implications for Cold [(18)F]FDG-PET/CT Analyses. Mol Imaging Biol (2020) 22(2):425–33. 10.1007/s11307-019-01381-y 31147900

[27] Yoneshiro T Aita S Matsushita M Okamatsu-Ogura Y Kameya T Kawai Y. Age-related decrease in cold-activated brown adipose tissue and accumulation of body fat in healthy humans. Obes (Silver Spring) (2011) 19(9):1755–60. 10.1038/oby.2011.125 21566561

[28] Pfannenberg C Werner MK Ripkens S Stef I Deckert A Schmadl M. Impact of age on the relationships of brown adipose tissue with sex and adiposity in humans. Diabetes (2010) 59(7):1789–93. 10.2337/db10-0004 PMC2889780 20357363

[29] Cedikova M Kripnerova M Dvorakova J Pitule P Grundmanova M Babuska V. Mitochondria in White, Brown, and Beige Adipocytes. Stem Cells Int (2016) 2016:6067349. 10.1155/2016/6067349 27073398 PMC4814709

[30] Bahler L Verberne HJ Admiraal WM Stok WJ Soeters MR Hoekstra JB. Differences in Sympathetic Nervous Stimulation of Brown Adipose Tissue Between the Young and Old, and the Lean and Obese. J Nucl Med (2016) 57(3):372–7. 10.2967/jnumed.115.165829 26609175

[31] Valle A Santandreu FM Garcia-Palmer FJ Roca P Oliver J. The serum levels of 17beta-estradiol, progesterone and triiodothyronine correlate with brown adipose tissue thermogenic parameters during aging. Cell Physiol Biochem (2008) 22(1-4):337–46. 10.1159/000149812 18769061

[32] Nedergaard J Cannon B. The changed metabolic world with human brown adipose tissue: therapeutic visions. Cell Metab (2010) 11(4):268–72. 10.1016/j.cmet.2010.03.007 20374959

[33] Justo R Frontera M Pujol E Rodriguez-Cuenca S Llado I Garcia-Palmer FJ. Gender-related differences in morphology and thermogenic capacity of brown adipose tissue mitochondrial subpopulations. Life Sci (2005) 76(10):1147–58. 10.1016/j.lfs.2004.08.019 15620578

[34] Rodriguez E Monjo M Rodriguez-Cuenca S Pujol E Amengual B Roca P. Sexual dimorphism in the adrenergic control of rat brown adipose tissue response to overfeeding. Pflugers Arch (2001) 442(3):396–403. 10.1007/s004240100556 11484771

[35] Quevedo S Roca P Pico C Palou A. Sex-associated differences in cold-induced UCP1 synthesis in rodent brown adipose tissue. Pflugers Arch (1998) 436(5):689–95. 10.1007/s004240050690 9716701

[36] Kaikaew K Steenbergen J van Dijk TH Grefhorst A Visser JA. Sex Difference in Corticosterone-Induced Insulin Resistance in Mice. Endocrinology (2019) 160(10):2367–87. 10.1210/en.2019-00194 PMC6760317 31265057

[37] Perkins AC Mshelia DS Symonds ME Sathekge M. Prevalence and pattern of brown adipose tissue distribution of 18F-FDG in patients undergoing PET-CT in a subtropical climatic zone. Nucl Med Commun (2013) 34(2):168–74. 10.1097/MNM.0b013e32835bbbf0 23196673

[38] Persichetti A Sciuto R Rea S Basciani S Lubrano C Mariani S. Prevalence, mass, and glucose-uptake activity of (1)(8)F-FDG-detected brown adipose tissue in humans living in a temperate zone of Italy. PloS One (2013) 8(5):e63391. 10.1371/journal.pone.0063391 23667608 PMC3648481

[39] Au-Yong IT Thorn N Ganatra R Perkins AC Symonds ME. Brown adipose tissue and seasonal variation in humans. Diabetes (2009) 58(11):2583–7. 10.2337/db09-0833 PMC2768171 19696186

[40] Saito M Okamatsu-Ogura Y Matsushita M Watanabe K Yoneshiro T Nio-Kobayashi J. High incidence of metabolically active brown adipose tissue in healthy adult humans: effects of cold exposure and adiposity. Diabetes (2009) 58(7):1526–31. 10.2337/db09-0530 PMC2699872 19401428

[41] Grefhorst A van den Beukel JC van Houten EL Steenbergen J Visser JA Themmen AP. Estrogens increase expression of bone morphogenetic protein 8b in brown adipose tissue of mice. Biol Sex Differ (2015) 6:7. 10.1186/s13293-015-0025-y 25866617 PMC4392498

[42] Thuzar M Law WP Ratnasingam J Jang C Dimeski G Ho KKY. Glucocorticoids suppress brown adipose tissue function in humans: A double-blind placebo-controlled study. Diabetes Obes Metab (2018) 20(4):840–8. 10.1111/dom.13157 29119718

[43] Kaikaew K Grefhorst A Steenbergen J Swagemakers SMA McLuskey A Visser JA. Sex difference in the mouse BAT transcriptome reveals a role of progesterone. J Mol Endocrinol (2021) 66(2):97–113. 10.1530/JME-20-0210 33263559

[44] Frontini A Vitali A Perugini J Murano I Romiti C Ricquier D. White-to-brown transdifferentiation of omental adipocytes in patients affected by pheochromocytoma. Biochim Biophys Acta (2013) 1831(5):950–9. 10.1016/j.bbalip.2013.02.005 23454374

[45] Berry DC Jiang Y Arpke RW Close EL Uchida A Reading D. Cellular Aging Contributes to Failure of Cold-Induced Beige Adipocyte Formation in Old Mice and Humans. Cell Metab (2017) 25(1):166–81. 10.1016/j.cmet.2016.10.023 PMC5226893 27889388

[46] Kotzbeck P Giordano A Mondini E Murano I Severi I Venema W. Brown adipose tissue whitening leads to brown adipocyte death and adipose tissue inflammation. J Lipid Res (2018) 59(5):784–94. 10.1194/jlr.M079665 PMC5928436 29599420

[47] Halberg N Khan T Trujillo ME Wernstedt-Asterholm I Attie AD Sherwani S. Hypoxia-inducible factor 1alpha induces fibrosis and insulin resistance in white adipose tissue. Mol Cell Biol (2009) 29(16):4467–83. 10.1128/MCB.00192-09 PMC2725728 19546236

[48] Kloting N Fasshauer M Dietrich A Kovacs P Schon MR Kern M. Insulin-sensitive obesity. Am J Physiol Endocrinol Metab (2010) 299(3):E506–15. 10.1152/ajpendo.00586.2009 20570822

[49] Lundgren M Svensson M Lindmark S Renstrom F Ruge T Eriksson JW. Fat cell enlargement is an independent marker of insulin resistance and ‘hyperleptinaemia’. Diabetologia (2007) 50(3):625–33. 10.1007/s00125-006-0572-1 17216279

[50] Salans LB Knittle JL Hirsch J. The role of adipose cell size and adipose tissue insulin sensitivity in the carbohydrate intolerance of human obesity. J Clin Invest (1968) 47(1):153–65. 10.1172/JCI105705 PMC297156 16695937

[51] Ghaben AL Scherer PE. Adipogenesis and metabolic health. Nat Rev Mol Cell Biol (2019) 20(4):242–58. 10.1038/s41580-018-0093-z 30610207

[52] Kloting N Bluher M. Adipocyte dysfunction, inflammation and metabolic syndrome. Rev Endocr Metab Disord (2014) 15(4):277–87. 10.1007/s11154-014-9301-0 25344447

[53] Fukumura D Ushiyama A Duda DG Xu L Tam J Krishna V. Paracrine regulation of angiogenesis and adipocyte differentiation during in vivo adipogenesis. Circ Res (2003) 93(9):e88–97. 10.1161/01.RES.0000099243.20096.FA PMC2755542 14525808

[54] Chusyd DE Wang D Huffman DM Nagy TR. Relationships between Rodent White Adipose Fat Pads and Human White Adipose Fat Depots. Front Nutr (2016) 3:10. 10.3389/fnut.2016.00010 27148535 PMC4835715

[55] Guglielmi V Sbraccia P. Obesity phenotypes: depot-differences in adipose tissue and their clinical implications. Eat Weight Disord (2018) 23(1):3–14. 10.1007/s40519-017-0467-9 29230714

[56] Durrer Schutz D Busetto L Dicker D Farpour-Lambert N Pryke R Toplak H. European Practical and Patient-Centred Guidelines for Adult Obesity Management in Primary Care. Obes Facts (2019) 12(1):40–66. 10.1159/000496183 30673677 PMC6465693

[57] Garvey WT Mechanick JI Brett EM Garber AJ Hurley DL Jastreboff AM. American Association of Clinical Endocrinologists and American College of Endocrinology Comprehensive Clinical Practice Guidelines for Medical Care of Patients with Obesity. Endocr Pract (2016) 22 Suppl 3:1–203. 10.4158/EP161365.GL 27219496

[58] Arnett DK Blumenthal RS Albert MA Buroker AB Goldberger ZD Hahn EJ. 2019 ACC/AHA Guideline on the Primary Prevention of Cardiovascular Disease: A Report of the American College of Cardiology/American Heart Association Task Force on Clinical Practice Guidelines. Circulation (2019) 140(11):e596–646. 10.1161/CIR.0000000000000678 PMC7734661 30879355

[59] Rosenzweig JL Bakris GL Berglund LF Hivert MF Horton ES Kalyani RR. Primary Prevention of ASCVD and T2DM in Patients at Metabolic Risk: An Endocrine Society* Clinical Practice Guideline. J Clin Endocrinol Metab (2019) 104(9):3939–85. 10.1210/jc.2019-01338 31365087

[60] Gallagher D Visser M Sepulveda D Pierson RN Harris T Heymsfield SB. How useful is body mass index for comparison of body fatness across age, sex, and ethnic groups? Am J Epidemiol (1996) 143(3):228–39. 10.1093/oxfordjournals.aje.a008733 8561156

[61] Kuk JL Lee S Heymsfield SB Ross R. Waist circumference and abdominal adipose tissue distribution: influence of age and sex. Am J Clin Nutr (2005) 81(6):1330–4. 10.1093/ajcn/81.6.1330 15941883

[62] Wells JC. Sexual dimorphism of body composition. Best Pract Res Clin Endocrinol Metab (2007) 21(3):415–30. 10.1016/j.beem.2007.04.007 17875489

[63] Karastergiou K Smith SR Greenberg AS Fried SK. Sex differences in human adipose tissues - the biology of pear shape. Biol Sex Differ (2012) 3(1):13. 10.1186/2042-6410-3-13 22651247 PMC3411490

[64] Palmer BF Clegg DJ. The sexual dimorphism of obesity. Mol Cell Endocrinol (2015) 402:113–9. 10.1016/j.mce.2014.11.029 PMC4326001 25578600

[65] Mauvais-Jarvis F Clegg DJ Hevener AL. The role of estrogens in control of energy balance and glucose homeostasis. Endocr Rev (2013) 34(3):309–38. 10.1210/er.2012-1055 PMC3660717 23460719

[66] Kautzky-Willer A Harreiter J Pacini G. Sex and Gender Differences in Risk, Pathophysiology and Complications of Type 2 Diabetes Mellitus. Endocr Rev (2016) 37(3):278–316. 10.1210/er.2015-1137 27159875 PMC4890267

[67] Demerath EW Sun SS Rogers N Lee M Reed D Choh AC. Anatomical patterning of visceral adipose tissue: race, sex, and age variation. Obes (Silver Spring) (2007) 15(12):2984–93. 10.1038/oby.2007.356 PMC2883307 18198307

[68] Macotela Y Boucher J Tran TT Kahn CR. Sex and depot differences in adipocyte insulin sensitivity and glucose metabolism. Diabetes (2009) 58(4):803–12. 10.2337/db08-1054 PMC2661589 19136652

[69] Schorr M Dichtel LE Gerweck AV Valera RD Torriani M Miller KK. Sex differences in body composition and association with cardiometabolic risk. Biol Sex Differ (2018) 9(1):28. 10.1186/s13293-018-0189-3 29950175 PMC6022328

[70] Taylor RW Grant AM Williams SM Goulding A. Sex differences in regional body fat distribution from pre- to postpuberty. Obes (Silver Spring) (2010) 18(7):1410–6. 10.1038/oby.2009.399 19893501

[71] Camhi SM Bray GA Bouchard C Greenway FL Johnson WD Newton RL. The relationship of waist circumference and BMI to visceral, subcutaneous, and total body fat: sex and race differences. Obes (Silver Spring) (2011) 19(2):402–8. 10.1038/oby.2010.248 PMC3960785 20948514

[72] Svendsen OL Hassager C Christiansen C. Age- and menopause-associated variations in body composition and fat distribution in healthy women as measured by dual-energy X-ray absorptiometry. Metabolism (1995) 44(3):369–73. 10.1016/0026-0495(95)90168-x 7885283

[73] Newell-Fugate AE. The role of sex steroids in white adipose tissue adipocyte function. Reproduction (2017) 153(4):R133–49. 10.1530/REP-16-0417 28115579

[74] Morrison SF Nakamura K. Central Mechanisms for Thermoregulation. Annu Rev Physiol (2019) 81:285–308. 10.1146/annurev-physiol-020518-114546 30256726

[75] Blondin DP Labbe SM Tingelstad HC Noll C Kunach M Phoenix S. Increased brown adipose tissue oxidative capacity in cold-acclimated humans. J Clin Endocrinol Metab (2014) 99(3):E438–46. 10.1210/jc.2013-3901 PMC4213359 24423363

[76] Cannon B Nedergaard J. Metabolic consequences of the presence or absence of the thermogenic capacity of brown adipose tissue in mice (and probably in humans). Int J Obes (Lond) (2010) 34 Suppl 1:S7–16. 10.1038/ijo.2010.177 20935668

[77] Blondin DP Nielsen S Kuipers EN Severinsen MC Jensen VH Miard S. Human Brown Adipocyte Thermogenesis Is Driven by beta2-AR Stimulation. Cell Metab (2020) 32(2):287–300 e7. 10.1016/j.cmet.2020.07.005 32755608

[78] Cypess AM Weiner LS Roberts-Toler C Franquet Elia E Kessler SH Kahn PA. Activation of human brown adipose tissue by a beta3-adrenergic receptor agonist. Cell Metab (2015) 21(1):33–8. 10.1016/j.cmet.2014.12.009 PMC4298351 25565203

[79] Okuyama C Kikuchi R Ikeuchi T. FDG Uptake in Brown Adipose Tissue Activated by a beta3-Adrenergic Receptor Agonist Prescribed for Overactive Bladder. Clin Nucl Med (2020) 45(8):628–31. 10.1097/RLU.0000000000003078 32453085

[80] Li Y Fromme T Schweizer S Schottl T Klingenspor M. Taking control over intracellular fatty acid levels is essential for the analysis of thermogenic function in cultured primary brown and brite/beige adipocytes. EMBO Rep (2014) 15(10):1069–76. 10.15252/embr.201438775 PMC4253847 25135951

[81] Labbe SM Caron A Bakan I Laplante M Carpentier AC Lecomte R. In vivo measurement of energy substrate contribution to cold-induced brown adipose tissue thermogenesis. FASEB J (2015) 29(5):2046–58. 10.1096/fj.14-266247 25681456

[82] Haemmerle G Lass A Zimmermann R Gorkiewicz G Meyer C Rozman J. Defective lipolysis and altered energy metabolism in mice lacking adipose triglyceride lipase. Science (5774) 2006) 312:734–7. 10.1126/science.1123965 16675698

[83] Kalinovich AV de Jong JM Cannon B Nedergaard J. UCP1 in adipose tissues: two steps to full browning. Biochimie (2017) 134:127–37. 10.1016/j.biochi.2017.01.007 28109720

[84] Evans BA Merlin J Bengtsson T Hutchinson DS. Adrenoceptors in white, brown, and brite adipocytes. Br J Pharmacol (2019) 176(14):2416–32. 10.1111/bph.14631 PMC6592855 30801689

[85] Hao Q Yadav R Basse AL Petersen S Sonne SB Rasmussen S. Transcriptome profiling of brown adipose tissue during cold exposure reveals extensive regulation of glucose metabolism. Am J Physiol Endocrinol Metab (2015) 308(5):E380–92. 10.1152/ajpendo.00277.2014 25516548

[86] Wang W Kissig M Rajakumari S Huang L Lim HW Won KJ. Ebf2 is a selective marker of brown and beige adipogenic precursor cells. Proc Natl Acad Sci USA (2014) 111(40):14466–71. 10.1073/pnas.1412685111 PMC4209986 25197048

[87] Barbatelli G Murano I Madsen L Hao Q Jimenez M Kristiansen K. The emergence of cold-induced brown adipocytes in mouse white fat depots is determined predominantly by white to brown adipocyte transdifferentiation. Am J Physiol Endocrinol Metab (2010) 298(6):E1244–53. 10.1152/ajpendo.00600.2009 20354155

[88] Finlin BS Memetimin H Confides AL Kasza I Zhu B Vekaria HJ. Human adipose beiging in response to cold and mirabegron. JCI Insight (2018) 3(15):e121510. 10.1172/jci.insight.121510 PMC6129119 30089732

[89] de Jong JM Larsson O Cannon B Nedergaard J. A stringent validation of mouse adipose tissue identity markers. Am J Physiol Endocrinol Metab (2015) 308(12):E1085–105. 10.1152/ajpendo.00023.2015 25898951

[90] Scheele C Wolfrum C. Brown Adipose Crosstalk in Tissue Plasticity and Human Metabolism. Endocr Rev (2020) 41(1):53–65. 10.1210/endrev/bnz007 PMC7006230 31638161

[91] Liu X Zheng Z Zhu X Meng M Li L Shen Y. Brown adipose tissue transplantation improves whole-body energy metabolism. Cell Res (2013) 23(6):851–4. 10.1038/cr.2013.64 PMC3674396 23649313

[92] Stanford KI Middelbeek RJ Townsend KL An D Nygaard EB Hitchcox KM. Brown adipose tissue regulates glucose homeostasis and insulin sensitivity. J Clin Invest (2013) 123(1):215–23. 10.1172/JCI62308 PMC3533266 23221344

[93] Maurer SF Fromme T Mocek S Zimmermann A Klingenspor M. Uncoupling protein 1 and the capacity for nonshivering thermogenesis are components of the glucose homeostatic system. Am J Physiol Endocrinol Metab (2020) 318(2):E198–215. 10.1152/ajpendo.00121.2019 31714796

[94] Villarroya J Cereijo R Gavalda-Navarro A Peyrou M Giralt M Villarroya F. New insights into the secretory functions of brown adipose tissue. J Endocrinol (2019) 243(2):R19–27. 10.1530/JOE-19-0295 31419785

[95] Laycock J Meeran K. The Gonads (1): Testes and The Ganads (2): Ovaries. In: Integrated Endocrinology. West Sussex: John Wiley & Sons (2013). p. 133–88.

[96] Zimmerman MA Budish RA Kashyap S Lindsey SH. GPER-novel membrane oestrogen receptor. Clin Sci (Lond) (2016) 130(12):1005–16. 10.1042/CS20160114 PMC5125080 27154744

[97] Garg D Ng SSM Baig KM Driggers P Segars J. Progesterone-Mediated Non-Classical Signaling. Trends Endocrinol Metab (2017) 28(9):656–68. 10.1016/j.tem.2017.05.006 28651856

[98] Navarro G Allard C Xu W Mauvais-Jarvis F. The role of androgens in metabolism, obesity, and diabetes in males and females. Obes (Silver Spring) (2015) 23(4):713–9. 10.1002/oby.21033 PMC4380643 25755205

[99] Miller WL Auchus RJ. The molecular biology, biochemistry, and physiology of human steroidogenesis and its disorders. Endocr Rev (2011) 32(1):81–151. 10.1210/er.2010-0013 21051590 PMC3365799

[100] Wake DJ Strand M Rask E Westerbacka J Livingstone DE Soderberg S. Intra-adipose sex steroid metabolism and body fat distribution in idiopathic human obesity. Clin Endocrinol (Oxf) (2007) 66(3):440–6. 10.1111/j.1365-2265.2007.02755.x 17302881

[101] Tchernof A Mansour MF Pelletier M Boulet MM Nadeau M Luu-The V. Updated survey of the steroid-converting enzymes in human adipose tissues. J Steroid Biochem Mol Biol (2015) 147:56–69. 10.1016/j.jsbmb.2014.11.011 25448733

[102] Ohlsson C Hammarstedt A Vandenput L Saarinen N Ryberg H Windahl SH. Increased adipose tissue aromatase activity improves insulin sensitivity and reduces adipose tissue inflammation in male mice. Am J Physiol Endocrinol Metab (2017) 313(4):E450–E62. 10.1152/ajpendo.00093.2017 PMC5668598 28655716

[103] Zhao H Innes J Brooks DC Reierstad S Yilmaz MB Lin Z. A novel promoter controls Cyp19a1 gene expression in mouse adipose tissue. Reprod Biol Endocrinol (2009) 7:37. 10.1186/1477-7827-7-37 19393092 PMC2684739

[104] Kim NR David K Corbeels K Khalil R Antonio L Schollaert D. Testosterone reduces body fat in male mice by stimulation of physical activity via extrahypothalamic ERalpha signaling. Endocrinology (2021). 10.1210/endocr/bqab045 PMC8140602 33674833

[105] Sharp LZ Shinoda K Ohno H Scheel DW Tomoda E Ruiz L. Human BAT possesses molecular signatures that resemble beige/brite cells. PloS One (2012) 7(11):e49452. 10.1371/journal.pone.0049452 23166672 PMC3500293

[106] Rodriguez-Cuenca S Monjo M Frontera M Gianotti M Proenza AM Roca P. Sex steroid receptor expression profile in brown adipose tissue. Effects of hormonal status. Cell Physiol Biochem (2007) 20(6):877–86. 10.1159/000110448 17982270

[107] Kroon J Pereira AM Meijer OC. Glucocorticoid Sexual Dimorphism in Metabolism: Dissecting the Role of Sex Hormones. Trends Endocrinol Metab (2020) 31(5):357–67. 10.1016/j.tem.2020.01.010 32037025

[108] Granner DK Wang JC Yamamoto KR. Regulatory Actions of Glucocorticoid Hormones: From Organisms to Mechanisms. Adv Exp Med Biol (2015) 872:3–31. 10.1007/978-1-4939-2895-8_1 26215988

[109] Pasquali R Vicennati V Cacciari M Pagotto U. The hypothalamic-pituitary-adrenal axis activity in obesity and the metabolic syndrome. Ann N Y Acad Sci (2006) 1083:111–28. 10.1196/annals.1367.009 17148736

[110] Heck AL Handa RJ. Sex differences in the hypothalamic-pituitary-adrenal axis’ response to stress: an important role for gonadal hormones. Neuropsychopharmacology (2019) 44(1):45–58. 10.1038/s41386-018-0167-9 30111811 PMC6235871

[111] Quarta C Mazza R Pasquali R Pagotto U. Role of sex hormones in modulation of brown adipose tissue activity. J Mol Endocrinol (2012) 49(1):R1–7. 10.1530/JME-12-0043 22460126

[112] Yoshioka K Yoshida T Wakabayashi Y Nishioka H Kondo M. Reduced brown adipose tissue thermogenesis of obese rats after ovariectomy. Endocrinol Jpn (1988) 35(4):537–43. 10.1507/endocrj1954.35.537 2850906

[113] Pedersen SB Bruun JM Kristensen K Richelsen B. Regulation of UCP1, UCP2, and UCP3 mRNA expression in brown adipose tissue, white adipose tissue, and skeletal muscle in rats by estrogen. Biochem Biophys Res Commun (2001) 288(1):191–7. 10.1006/bbrc.2001.5763 11594772

[114] Martinez de Morentin PB Gonzalez-Garcia I Martins L Lage R Fernandez-Mallo D Martinez-Sanchez N. Estradiol regulates brown adipose tissue thermogenesis via hypothalamic AMPK. Cell Metab (2014) 20(1):41–53. 10.1016/j.cmet.2014.03.031 24856932 PMC4082097

[115] Monjo M Rodriguez AM Palou A Roca P. Direct effects of testosterone, 17 beta-estradiol, and progesterone on adrenergic regulation in cultured brown adipocytes: potential mechanism for gender-dependent thermogenesis. Endocrinology (2003) 144(11):4923–30. 10.1210/en.2003-0537 12959998

[116] Rodriguez-Cuenca S Monjo M Gianotti M Proenza AM Roca P. Expression of mitochondrial biogenesis-signaling factors in brown adipocytes is influenced specifically by 17beta-estradiol, testosterone, and progesterone. Am J Physiol Endocrinol Metab (2007) 292(1):E340–6. 10.1152/ajpendo.00175.2006 16954335

[117] Zhang W Schmull S Du M Liu J Lu Z Zhu H. Estrogen Receptor alpha and beta in Mouse: Adipose-Derived Stem Cell Proliferation, Migration, and Brown Adipogenesis In Vitro. Cell Physiol Biochem (2016) 38(6):2285–99. 10.1159/000445583 27197672

[118] Heine PA Taylor JA Iwamoto GA Lubahn DB Cooke PS. Increased adipose tissue in male and female estrogen receptor-alpha knockout mice. Proc Natl Acad Sci USA (2000) 97(23):12729–34. 10.1073/pnas.97.23.12729 PMC18832 11070086

[119] Zhou Z Moore TM Drew BG Ribas V Wanagat J Civelek M. Estrogen receptor alpha controls metabolism in white and brown adipocytes by regulating Polg1 and mitochondrial remodeling. Sci Transl Med (2020) 12(555):eaax8096. 10.1126/scitranslmed.aax8096 32759275 PMC8212422

[120] Ohlsson C Hellberg N Parini P Vidal O Bohlooly YM Rudling M. Obesity and disturbed lipoprotein profile in estrogen receptor-alpha-deficient male mice. Biochem Biophys Res Commun (2000) 278(3):640–5. 10.1006/bbrc.2000.3827 11095962

[121] Gonzalez-Granillo M Savva C Li X Fitch M Pedrelli M Hellerstein M. ERbeta activation in obesity improves whole body metabolism via adipose tissue function and enhanced mitochondria biogenesis. Mol Cell Endocrinol (2019) 479:147–58. 10.1016/j.mce.2018.10.007 30342056

[122] Ponnusamy S Tran QT Harvey I Smallwood HS Thiyagarajan T Banerjee S. Pharmacologic activation of estrogen receptor beta increases mitochondrial function, energy expenditure, and brown adipose tissue. FASEB J (2017) 31(1):266–81. 10.1096/fj.201600787RR PMC5161516 27733447

[123] Davis KE Carstens EJ Irani BG Gent LM Hahner LM Clegg DJ. Sexually dimorphic role of G protein-coupled estrogen receptor (GPER) in modulating energy homeostasis. Horm Behav (2014) 66(1):196–207. 10.1016/j.yhbeh.2014.02.004 24560890 PMC4051842

[124] Rodriguez AM Monjo M Roca P Palou A. Opposite actions of testosterone and progesterone on UCP1 mRNA expression in cultured brown adipocytes. Cell Mol Life Sci (2002) 59(10):1714–23. 10.1007/pl00012499 PMC11337545 12475182

[125] Xu Y Lopez M. Central regulation of energy metabolism by estrogens. Mol Metab (2018) 15:104–15. 10.1016/j.molmet.2018.05.012 PMC6066788 29886181

[126] Yonezawa R Wada T Matsumoto N Morita M Sawakawa K Ishii Y. Central versus peripheral impact of estradiol on the impaired glucose metabolism in ovariectomized mice on a high-fat diet. Am J Physiol Endocrinol Metab (2012) 303(4):E445–56. 10.1152/ajpendo.00638.2011 22550066

[127] McIlvride S Mushtaq A Papacleovoulou G Hurling C Steel J Jansen E. A progesterone-brown fat axis is involved in regulating fetal growth. Sci Rep (2017) 7(1):10671. 10.1038/s41598-017-10979-7 28878263 PMC5587669

[128] Cannon B Nedergaard J. Brown adipose tissue: function and physiological significance. Physiol Rev (2004) 84(1):277–359. 10.1152/physrev.00015.2003 14715917

[129] Fuller-Jackson JP Dordevic AL Clarke IJ Henry BA. Effect of sex and sex steroids on brown adipose tissue heat production in humans. Eur J Endocrinol (2020) 183(3):343–55. 10.1530/EJE-20-0184 32508310

[130] Zennaro MC Le Menuet D Viengchareun S Walker F Ricquier D Lombes M. Hibernoma development in transgenic mice identifies brown adipose tissue as a novel target of aldosterone action. J Clin Invest (1998) 101(6):1254–60. 10.1172/JCI1915 PMC508679 9502766

[131] Furuhata R Kabe Y Kanai A Sugiura Y Tsugawa H Sugiyama E. Progesterone receptor membrane associated component 1 enhances obesity progression in mice by facilitating lipid accumulation in adipocytes. Commun Biol (2020) 3(1):479. 10.1038/s42003-020-01202-x 32887925 PMC7473863

[132] Galmozzi A Kok BP Kim AS Montenegro-Burke JR Lee JY Spreafico R. PGRMC2 is an intracellular haem chaperone critical for adipocyte function. Nature (2019) 576(7785):138–42. 10.1038/s41586-019-1774-2 PMC6895438 31748741

[133] Lerner A Kewada D Ahmed A Hardy K Christian M Franks S. Androgen Reduces Mitochondrial Respiration in Mouse Brown Adipocytes: A Model for Disordered Energy Balance in Polycystic Ovary Syndrome. Int J Mol Sci (2020) 22(1):243. 10.3390/ijms22010243 PMC7796281 33383677

[134] Hashimoto O Noda T Morita A Morita M Ohtsuki H Sugiyama M. Castration induced browning in subcutaneous white adipose tissue in male mice. Biochem Biophys Res Commun (2016) 478(4):1746–50. 10.1016/j.bbrc.2016.09.017 27608598

[135] Gasparini SJ Swarbrick MM Kim S Thai LJ Henneicke H Cavanagh LL. Androgens sensitise mice to glucocorticoid-induced insulin resistance and fat accumulation. Diabetologia (2019) 62(8):1463–77. 10.1007/s00125-019-4887-0 31098671

[136] Moverare-Skrtic S Venken K Andersson N Lindberg MK Svensson J Swanson C. Dihydrotestosterone treatment results in obesity and altered lipid metabolism in orchidectomized mice. Obes (Silver Spring) (2006) 14(4):662–72. 10.1038/oby.2006.75 16741268

[137] Nohara K Laque A Allard C Munzberg H Mauvais-Jarvis F. Central mechanisms of adiposity in adult female mice with androgen excess. Obes (Silver Spring) (2014) 22(6):1477–84. 10.1002/oby.20719 PMC4037375 24639082

[138] Spaanderman DCE Nixon M Buurstede JC Sips HC Schilperoort M Kuipers EN. Androgens modulate glucocorticoid receptor activity in adipose tissue and liver. J Endocrinol (2018) 240(1):51–63. 10.1530/JOE-18-0503 30400038

[139] Fan W Yanase T Nomura M Okabe T Goto K Sato T. Androgen receptor null male mice develop late-onset obesity caused by decreased energy expenditure and lipolytic activity but show normal insulin sensitivity with high adiponectin secretion. Diabetes (2005) 54(4):1000–8. 10.2337/diabetes.54.4.1000 15793238

[140] Escobar-Morreale HF Alvarez-Blasco F Botella-Carretero JI Luque-Ramirez M. The striking similarities in the metabolic associations of female androgen excess and male androgen deficiency. Hum Reprod (2014) 29(10):2083–91. 10.1093/humrep/deu198 25104855

[141] Shorakae S Jona E de Courten B Lambert GW Lambert EA Phillips SE. Brown adipose tissue thermogenesis in polycystic ovary syndrome. Clin Endocrinol (Oxf) (2019) 90(3):425–32. 10.1111/cen.13913 30548504

[142] Benrick A Chanclon B Micallef P Wu Y Hadi L Shelton JM. Adiponectin protects against development of metabolic disturbances in a PCOS mouse model. Proc Natl Acad Sci USA (2017) 114(34):E7187–E96. 10.1073/pnas.1708854114 PMC5576831 28790184

[143] Yuan X Hu T Zhao H Huang Y Ye R Lin J. Brown adipose tissue transplantation ameliorates polycystic ovary syndrome. Proc Natl Acad Sci USA (2016) 113(10):2708–13. 10.1073/pnas.1523236113 PMC4790997 26903641

[144] Lin HY Xu Q Yeh S Wang RS Sparks JD Chang C. Insulin and leptin resistance with hyperleptinemia in mice lacking androgen receptor. Diabetes (2005) 54(6):1717–25. 10.2337/diabetes.54.6.1717 15919793

[145] Stocco C. Tissue physiology and pathology of aromatase. Steroids (2012) 77(1-2):27–35. 10.1016/j.steroids.2011.10.013 22108547 PMC3286233

[146] van den Beukel JC Boon MR Steenbergen J Rensen PC Meijer OC Themmen AP. Cold Exposure Partially Corrects Disturbances in Lipid Metabolism in a Male Mouse Model of Glucocorticoid Excess. Endocrinology (2015) 156(11):4115–28. 10.1210/en.2015-1092 26372178

[147] van den Beukel JC Grefhorst A Quarta C Steenbergen J Mastroberardino PG Lombes M. Direct activating effects of adrenocorticotropic hormone (ACTH) on brown adipose tissue are attenuated by corticosterone. FASEB J (2014) 28(11):4857–67. 10.1096/fj.14-254839 25085924

[148] Mousovich-Neto F Matos MS Costa ACR de Melo Reis RA Atella GC Miranda-Alves L. Brown adipose tissue remodelling induced by corticosterone in male Wistar rats. Exp Physiol (2019) 104(4):514–28. 10.1113/EP087332 30653762

[149] Soumano K Desbiens S Rabelo R Bakopanos E Camirand A Silva JE. Glucocorticoids inhibit the transcriptional response of the uncoupling protein-1 gene to adrenergic stimulation in a brown adipose cell line. Mol Cell Endocrinol (2000) 165(1-2):7–15. 10.1016/s0303-7207(00)00276-8 10940478

[150] Kroon J Koorneef LL van den Heuvel JK Verzijl CRC van de Velde NM Mol IM. Selective Glucocorticoid Receptor Antagonist CORT125281 Activates Brown Adipose Tissue and Alters Lipid Distribution in Male Mice. Endocrinology (2018) 159(1):535–46. 10.1210/en.2017-00512 28938459

[151] Moriscot A Rabelo R Bianco AC. Corticosterone inhibits uncoupling protein gene expression in brown adipose tissue. Am J Physiol (1993) 265(1 Pt 1):E81–7. 10.1152/ajpendo.1993.265.1.E81 8338156

[152] Koorneef LL Kroon J Viho EMG Wahl LF Heckmans KML van Dorst M. The selective glucocorticoid receptor antagonist CORT125281 has tissue-specific activity. J Endocrinol (2020) 246(1):79–92. 10.1530/JOE-19-0486 32369774 PMC7274539

[153] Penfornis P Viengchareun S Le Menuet D Cluzeaud F Zennaro MC Lombes M. The mineralocorticoid receptor mediates aldosterone-induced differentiation of T37i cells into brown adipocytes. Am J Physiol Endocrinol Metab (2000) 279(2):E386–94. 10.1152/ajpendo.2000.279.2.E386 10913039

[154] Viengchareun S Penfornis P Zennaro MC Lombes M. Mineralocorticoid and glucocorticoid receptors inhibit UCP expression and function in brown adipocytes. Am J Physiol Endocrinol Metab (2001) 280(4):E640–9. 10.1152/ajpendo.2001.280.4.E640 11254472

[155] Ferguson D Hutson I Tycksen E Pietka TA Bauerle K Harris CA. Role of Mineralocorticoid Receptor in Adipogenesis and Obesity in Male Mice. Endocrinology (2020) 161(2):bqz010. 10.1210/endocr/bqz010 32036385 PMC7007880

[156] Armani A Cinti F Marzolla V Morgan J Cranston GA Antelmi A. Mineralocorticoid receptor antagonism induces browning of white adipose tissue through impairment of autophagy and prevents adipocyte dysfunction in high-fat-diet-fed mice. FASEB J (2014) 28(8):3745–57. 10.1096/fj.13-245415 24806198

[157] Thuzar M Law WP Dimeski G Stowasser M Ho KKY. Mineralocorticoid antagonism enhances brown adipose tissue function in humans: A randomized placebo-controlled cross-over study. Diabetes Obes Metab (2019) 21(3):509–16. 10.1111/dom.13539 30225967

[158] Luijten IHN Brooks K Boulet N Shabalina IG Jaiprakash A Carlsson B. Glucocorticoid-Induced Obesity Develops Independently of UCP1. Cell Rep (2019) 27(6):1686–98 e5. 10.1016/j.celrep.2019.04.041 31067456

[159] Glantschnig C Mattijssen F Vogl ES Ali Khan A Rios Garcia M Fischer K. The glucocorticoid receptor in brown adipocytes is dispensable for control of energy homeostasis. EMBO Rep (2019) 20(11):e48552. 10.15252/embr.201948552 31559673 PMC6832000

[160] Vander Tuig JG Ohshima K Yoshida T Romsos DR Bray GA. Adrenalectomy increases norepinephrine turnover in brown adipose tissue of obese (ob/ob) mice. Life Sci (1984) 34(15):1423–32. 10.1016/0024-3205(84)90056-0 6708740

[161] van den Berg R Kooijman S Noordam R Ramkisoensing A Abreu-Vieira G Tambyrajah LL. A Diurnal Rhythm in Brown Adipose Tissue Causes Rapid Clearance and Combustion of Plasma Lipids at Wakening. Cell Rep (2018) 22(13):3521–33. 10.1016/j.celrep.2018.03.004 29590620

[162] Nieman LK Ilias I. Evaluation and treatment of Cushing’s syndrome. Am J Med (2005) 118(12):1340–6. 10.1016/j.amjmed.2005.01.059 16378774

[163] Ramage LE Akyol M Fletcher AM Forsythe J Nixon M Carter RN. Glucocorticoids Acutely Increase Brown Adipose Tissue Activity in Humans, Revealing Species-Specific Differences in UCP-1 Regulation. Cell Metab (2016) 24(1):130–41. 10.1016/j.cmet.2016.06.011 PMC4949380 27411014

[164] Scotney H Symonds ME Law J Budge H Sharkey D Manolopoulos KN. Glucocorticoids modulate human brown adipose tissue thermogenesis in vivo. Metabolism (2017) 70:125–32. 10.1016/j.metabol.2017.01.024 PMC5395593 28403937

[165] Pereira CD Azevedo I Monteiro R Martins MJ. 11beta-Hydroxysteroid dehydrogenase type 1: relevance of its modulation in the pathophysiology of obesity, the metabolic syndrome and type 2 diabetes mellitus. Diabetes Obes Metab (2012) 14(10):869–81. 10.1111/j.1463-1326.2012.01582.x 22321826

[166] Chapman K Holmes M Seckl J. 11beta-hydroxysteroid dehydrogenases: intracellular gate-keepers of tissue glucocorticoid action. Physiol Rev (2013) 93(3):1139–206. 10.1152/physrev.00020.2012 PMC3962546 23899562

[167] Doig CL Fletcher RS Morgan SA McCabe EL Larner DP Tomlinson JW. 11beta-HSD1 Modulates the Set Point of Brown Adipose Tissue Response to Glucocorticoids in Male Mice. Endocrinology (2017) 158(6):1964–76. 10.1210/en.2016-1722 PMC5460930 28368470

[168] Liu J Kong X Wang L Qi H Di W Zhang X. Essential roles of 11beta-HSD1 in regulating brown adipocyte function. J Mol Endocrinol (2013) 50(1):103–13. 10.1530/JME-12-0099 23197361

[169] Bangasser DA Valentino RJ. Sex differences in stress-related psychiatric disorders: neurobiological perspectives. Front Neuroendocrinol (2014) 35(3):303–19. 10.1016/j.yfrne.2014.03.008 PMC4087049 24726661

[170] Kitay JI. Pituitary-Adrenal Function in the Rat after Gonadectomy and Gonadal Hormone Replacement. Endocrinology (1963) 73:253–60. 10.1210/endo-73-2-253 14076206

[171] Seale JV Wood SA Atkinson HC Bate E Lightman SL Ingram CD. Gonadectomy reverses the sexually diergic patterns of circadian and stress-induced hypothalamic-pituitary-adrenal axis activity in male and female rats. J Neuroendocrinol (2004) 16(6):516–24. 10.1111/j.1365-2826.2004.01195.x 15189326

[172] Ruiz D Padmanabhan V Sargis RM. Stress, Sex, and Sugar: Glucocorticoids and Sex-Steroid Crosstalk in the Sex-Specific Misprogramming of Metabolism. J Endocr Soc bvaa087 (2020) 4(8):bvaa087. 10.1210/jendso/bvaa087 32734132 PMC7382384

[173] Dieudonne MN Sammari A Dos Santos E Leneveu MC Giudicelli Y Pecquery R. Sex steroids and leptin regulate 11beta-hydroxysteroid dehydrogenase I and P450 aromatase expressions in human preadipocytes: Sex specificities. J Steroid Biochem Mol Biol (2006) 99(4-5):189–96. 10.1016/j.jsbmb.2006.01.007 16621515

[174] Andersson T Simonyte K Andrew R Strand M Buren J Walker BR. Tissue-specific increases in 11beta-hydroxysteroid dehydrogenase type 1 in normal weight postmenopausal women. PloS One (2009) 4(12):e8475. 10.1371/journal.pone.0008475 20041117 PMC2795198

[175] Mauvais-Jarvis F Arnold AP Reue K. A Guide for the Design of Pre-clinical Studies on Sex Differences in Metabolism. Cell Metab (2017) 25(6):1216–30. 10.1016/j.cmet.2017.04.033 PMC5516948 28591630

[176] van den Beukel JC Grefhorst A Hoogduijn MJ Steenbergen J Mastroberardino PG Dor FJ. Women have more potential to induce browning of perirenal adipose tissue than men. Obes (Silver Spring) (2015) 23(8):1671–9. 10.1002/oby.21166 26179979

[177] Witchel SF. Disorders of sex development. Best Pract Res Clin Obstet Gynaecol (2018) 48:90–102. 10.1016/j.bpobgyn.2017.11.005 29503125 PMC5866176

[178] Chen X McClusky R Chen J Beaven SW Tontonoz P Arnold AP. The number of x chromosomes causes sex differences in adiposity in mice. PloS Genet (2012) 8(5):e1002709. 10.1371/journal.pgen.1002709 22589744 PMC3349739

[179] Sambeat A Gulyaeva O Dempersmier J Sul HS. Epigenetic Regulation of the Thermogenic Adipose Program. Trends Endocrinol Metab (2017) 28(1):19–31. 10.1016/j.tem.2016.09.003 27692461 PMC5183481

[180] Chu SH Loucks EB Kelsey KT Gilman SE Agha G Eaton CB. Sex-specific epigenetic mediators between early life social disadvantage and adulthood BMI. Epigenomics (2018) 10(6):707–22. 10.2217/epi-2017-0146 PMC6367732 29888956

[181] Jang H Bhasin S Guarneri T Serra C Schneider M Lee MJ. The Effects of a Single Developmentally Entrained Pulse of Testosterone in Female Neonatal Mice on Reproductive and Metabolic Functions in Adult Life. Endocrinology (2015) 156(10):3737–46. 10.1210/EN.2015-1117 PMC4588815 26132920

